# CloudForest: A Scalable and Efficient Random Forest Implementation for Biological Data

**DOI:** 10.1371/journal.pone.0144820

**Published:** 2015-12-17

**Authors:** Ryan Bressler, Richard B. Kreisberg, Brady Bernard, John E. Niederhuber, Joseph G. Vockley, Ilya Shmulevich, Theo A. Knijnenburg

**Affiliations:** 1 Institute for Systems Biology, Seattle, WA, United States of America; 2 Inova Translational Medicine Institute, Inova Health System and Inova Fairfax Medical Center, Falls Church, VA, United States of America; 3 Virginia Commonwealth University, School of Medicine, Richmond, VA, United States of America; University of Pittsburgh, UNITED STATES

## Abstract

Random Forest has become a standard data analysis tool in computational biology. However, extensions to existing implementations are often necessary to handle the complexity of biological datasets and their associated research questions. The growing size of these datasets requires high performance implementations. We describe CloudForest, a Random Forest package written in Go, which is particularly well suited for large, heterogeneous, genetic and biomedical datasets. CloudForest includes several extensions, such as dealing with unbalanced classes and missing values. Its flexible design enables users to easily implement additional extensions. CloudForest achieves fast running times by effective use of the CPU cache, optimizing for different classes of features and efficiently multi-threading. https://github.com/ilyalab/CloudForest.

## Introduction

Random Forest (RF) [1] has become a widely-used method for classification and regression analysis of biological data. It often achieves good prediction performance on datasets that are characterized by a large number of features and a relatively small number of samples [2]. For example, Random Forest consistently performs very well in the DREAM prediction challenges [3].

Importantly, the best performance on biological data is generally not achieved with the standard Random Forest implementation. Specific extensions and adaptations have been developed to handle the intricacies of certain biological datasets and associated research questions [4, 5]. These include, but are not limited to: unbalanced classes, heterogeneous feature types, alternative notions of feature importance, methods for feature selection, and robustness against noisy and missing data. Additionally, the huge number of features in biological datasets that are derived from high-throughput genome-wide measurement technologies, such as microarrays and sequencing platforms, necessitates fast RF implementations.

We developed CloudForest, a well-documented RF package (see S1 File) with a flexible design that enables straightforward implementation of extensions, many of which are already present in the current version. Here, we describe the underlying structure and features of CloudForest and compare it to the most widely used RF packages in terms of prediction performance and computation time.

## 1 Methods

CloudForest has been written in Go (http://golang.org/), a language developed at Google that strives to balance the speed of a low-level compiled language with the ease of development of a higher level language. Specifically, Go code can achieve speeds near that of compiled languages like C while allowing relatively terse code, which is familiar to programmers accustomed to scripting languages like Python or R. Go supports functional programing paradigms that map well to operations with and on decision trees. See S1 File for some code snippets to illustrate this point.

CloudForest implements rigorously defined interfaces to represent splitting criteria and operations needed for split searching without restricting how the underlying data is represented. This enabled us to represent categorical and numerical data using the data type that is most efficient. As a result, CloudForest can natively handle categorical data types and missing values (Section 1.1). Additionally, CloudForest’s design accommodates rapid implementation of new extensions using the common core functionality. Some of the most useful extensions, such as dealing with unbalanced classes (Section 1.2) and alternative feature importance scores (Section 1.3), are already available. CloudForest’s design was prioritized to minimize training time by smart use of the CPU cache (Section 1.4) and efficient multi-threading (Section 1.5). CloudForest can be run from the command line and from a wrapper script written in an arbitrary programming language that pipes CloudForest commands directly to the terminal. In this way, CloudForest accommodates users that want to implement or experiment with RF extensions as well as users that simply want to use the existing functionalities in CloudForest. S1 File links to additional documentation to facilitate users that want to develop novel functionalities in CloudForest.

Table 1 shows a comparison between CloudForest and R’s randomForest package and scikit-learn’s RandomForestClassifier, two of the most widely use RF implementations.

**Table 1:** Overview of CloudForest’s main features compared with R’s randomForest package (R’s RF) and scikit-learn’s RandomForestClassifier (SKL RF).

	CloudForest	R’s RF	SKL RF
Implemented in	Go	Fortran, C, R	Cython
Handles categorial features	yes	yes	no
Handles missing values*	yes	no	no

*For all three approaches, missing values can be imputed.

### 1.1 Feature heterogeneity

Variables used in the RF can be of different types, i.e. numerical (either discrete or continuous) or categorical (ordinal or nominal), and features derived from biological measurements can fall into any of these types. For example, gene expression values are numerical variables. Features derived from sequencing data are often ordinal categorical, such as binary variant or mutations calls, or nominal categorical, such as zygosity calls. The distribution of values of these features in terms of sparsity and cardinality as well the number of missing values may differ dramatically across the feature set.

Features used in CloudForest can be encoded as numerical, (nominal) categorical and binary. The latter is a special case of ordinal categorical features. All other ordinal categorical features are considered numerical. CloudForest offers native support for fast split searching in categorical features via bits packed into integers as in Brieman’s Fortran implementation [6]. To support datasets with a large number of features CloudForest provides an alternative split search that uses Go’s big.Int to efficiently perform arithmetic with big integer numbers. Additionally, CloudForest implements a dedicated optimization for binary features and categorical features with a small cardinality. This enables faster and more efficient computation. CloudForest handles missing values natively using either a bias correction [7] or ‘three-way-splitting’ [8]. This is especially important for biological data, where missing values are very common.

### 1.2 Unbalanced classes

Many datasets in biology are unbalanced, meaning that there is a considerable difference in the number of samples per class. CloudForest implements several widely used approaches to deal with unbalanced classes, such as roughly balanced bagging of samples [9] and class-specific weighting of errors. Additionally, experimental boosting approaches, such as adaptive boosting [10], which often increase performance, are implemented as well.

### 1.3 Feature importance

Feature importance scores in RF are used as splitting criteria in the decision tree and are often useful as output to the user. Besides the commonly used Gini impurity and squared/L2 error, alternative scores can be quickly implemented. CloudForest includes additional impurity measures including entropy, weighted entropy, weighted Gini, absolute/L1 error and impurities based directly on misclassification cost. Additionally, CloudForest can output the global feature importance scores, mean-minimal-depth-used scores [11], the local importance scores and sample proximities [1].

Finally, CloudForest enables internal feature selection and feature importance significance testing via Artificial Contrasts with Ensembles (ACE) [12], which provides p-values for importance scores based on the comparison of feature performance to artificial contrasts features. This method has been shown to reduce bias towards high cardinality features. CloudForest further implements optional methods for vetting features during feature selection using out-of-bag samples or artificial contrasts.

### 1.4 Effective use of CPU cache

Training the multitude of decision trees that make up the RF requires, for each node in each tree, the selection of the best feature (from a randomly chosen set of features) and the value at which to split this feature. CloudForest employs data layouts and algorithms designed to maximize the efficacy of a modern CPU cache during split selection. The data for each individual feature within the data set is stored in a separate continuous array and all index and accumulator arrays are preallocated and reused within each thread. Inspired by an optimization to the internal sort used in split selection first introduced in scikit-learn v.15 [13], numerical values are copied into a preallocated, ordered array thereby accelerating performance by reducing value lookup time. Specifically, the sorting algorithm in CloudForest was optimized to minimize cache misses by ensuring that the data that need to be accessed during sorting are stored in continuous sections of memory that the operating system will recognize and place on the cache.

### 1.5 Multi-core CPU optimizations

RF training is embarrassingly parallel, since individual decision trees are learned independently. To support efficient multi-threading, CloudForest uses lock-free concurrent access to the underlying data where possible. It overcomes one of the main memory bottlenecks for RF by allowing forests to be written to disk as they are grown without needing to store the entire forest in memory. Additionally, the text based format used to store the trees can be easily concatenated, which allows the training of a single large forest to be parallelized across multiple machines. Importance scores and other statistics that must be gathered across the trees are calculated using thread safe data structures.

### 1.6 Ethics statement

Concerning the datasets described in Section 2.1: Individuals were recruited at Inova Fairfax Hospital during 2011–2013 and enrolled in the Inova Translational Medicine Institute’s clinical study entitled “Molecular Study of Pre-term Birth.” All study participants provided written informed consent for use of their genome sequences and medical records for research purposes. The “Molecular Study of Pre-term Birth” was approved by the Institutional Review Board of Inova Health System and the Western Institutional Review Board (#1124761). All patient data and information were anonymized and de-identified prior to analysis.

## 2 Results

We compared CloudForest to R’s randomForest package [14] and scikit-learn’s RandomForestClassifier [13]. R’s randomForest is based on Brieman’s original Fortran code and is the most established implementation. Scikit-learn’s implementation is in Cython (a compiled Python-like language) [15]. Scikit-learn v.15 offers one of the fastest available implementations on large numerical datasets.

### 2.1 Computation time and prediction performance

We applied these RF implementations on two large biomedical datasets (Table 2). The first dataset contains 738 clinical features derived from electronic medical records and patient surveys. They include numerical (44), binary (673) and categorical (21) features across a set of 659 samples (patients). The second dataset contains 167,698 genomic features for the same set of patient samples. These features are derived from whole-genome sequencing data, and include binary (87,084) and categorical (80,614) calls of homozygous and heterozygous minor allele variants. Both these datasets are part of an ongoing study on the causes of preterm birth. These datasets, initially described in [16], are examples of large and heterogeneous datasets, which characterize current data in biology. In particular, clinical features derived from electronic medical records and genomic features derived from sequence data often contain binary or categorical data.

**Table 2:** Overview of the two biomedical datasets used to evaluate CloudForest. The columns indicate (from left to right): the number of samples with the number of positives samples (targets) in parentheses, the number of numerical features, the number of binary features and the number of categorical features.

Dataset	# samples (targets)	# numerical f.	# binary f.	# categorial f.
clinical	659 (220)	44	673	21
genomic	659 (220)	0	87,084	80,614

The RF implementations were used to classify the 439 cases of fullterm birth and 220 cases of preterm birth, i.e. predict the class labels of these 659 samples. The clinical dataset was used to evaluate classification performance. To estimate the error, we employed a stratified 10-fold cross-validation scheme. The genomic dataset was used to evaluate the speed of the RF implementations.

Because R’s randomForest and scikit-learn do not handle missing values, we imputed all missing values to the feature mean for numerical features and feature mode for categorical features before analysis. Additionally, categorical data were encoded as numerical features for scikit-learn, as it does not support categorical data. This encoding was performed by creating a binary feature for each of the categories in the categorical feature. All implementations were set to grow 500 trees with their default parameters. Tests were conducted using a single thread, though both scikit-learn and CloudForest can efficiently do multi-threading.

**Fig 1:**
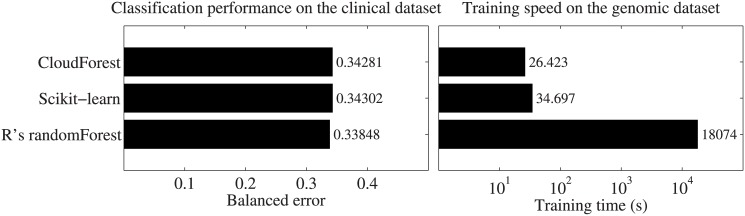
Comparison between CloudForest and other RF implementations in terms of prediction performance (**a**) and training time (**b**). The RFs consisted of 500 trees and were trained using the same standard parameter settings for all implementations.

The experiment demonstrated that CloudForest offers a classification performance equivalent to R and scikit-learn on the clinical dataset (Fig 1). However, it is faster than either implementation on the large heterogeneous genomic data set. Noteworthy, R is much slower than CloudForest and scikit-learn. The 24% speed-up with respect to scikit-learn is mainly due to CloudForest’s native ability to handle categorical features. If categorical features are made numerical, both approaches are comparable in terms of running time (Figure A in S1 File).

The extensions available in CloudForest, offer the user the ability to quickly evaluate various algorithmic variations on the RF. We observed that using roughly balanced bagging to handle the class unbalancedness substantially improved performance on the clinical dataset (Figure B in S1 File). Additional experiments on benchmark datasets from the LIBSVM data repository demonstrated that in some cases these extensions substantially lower error rates on independent test sets (Figure C in S1 File). These experiments also demonstrated that the training speed of scikit-learn and CloudForest are comparable.

### 2.2 Missing values

One prominent feature of CloudForest is its native support for missing values. We employed several datasets from The Cancer Genome Atlas (TCGA) to demonstrate the usefulness of this feature. Specifically, for each of 6 different tumor types, we created a large dataset containing numerical gene expression features, categorical and numerical copy number features and binary gene mutation features. Each dataset contains hundreds of samples (cancer patients) and thousands of features. We defined the classification task of predicting the mutation status of the often mutated tumor suppressor gene *TP53* using half of the samples for training and the other half for testing. In the training datasets, we artificially introduced missing values using a randomization scheme that preferentially puts missing values in features that are highly correlated with the target, i.e. the mutation status of *TP53* (See S1 File). This procedure was performed for increasing percentages of missing values in the dataset, and repeated 10 times.

**Fig 2:**
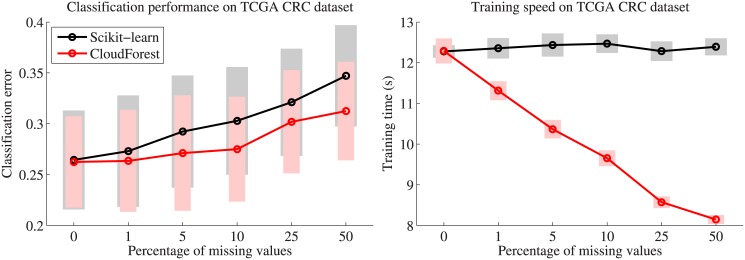
Comparison between CloudForest and scikit-learn in terms of prediction performance (**a**) and training time (**b**) for a TCGA dataset with varying numbers of missing values (x-axis). For scikit-learn missing values are imputed before RF analysis, whereas CloudForest natively handles missing values without imputation. The time necessary for imputation for scikit-learn is not included in the training times depicted.

We employed CloudForest and scikit-learn to train RFs on these training sets and test on the independent test set. For scikit-learn, which does not handle missing values, we imputed all missing values to the feature mean for numerical features and feature mode for categorical and binary features before analysis. Fig 2 depicts the results of this exercise for colorectal cancer (CRC), one of the six TCGA datasets. It is clear that CloudForest retains a consistently better prediction performance than scikit-learn, especially for the case of many missing values (Fig 2a). We found this pattern across most TCGA datasets, although not always as pronounced as for CRC (Figure D in S1 File). Moreover, the computation time to train the decision trees in CloudForest decreases with more missing values. This is not the case for scikit-learn, where missing values are imputed before RF analysis, leading to a dataset of the same size. Although the training speed of CloudForest and scikit-learn is similar for datasets without missing values, CloudForest is faster than scikit-learn for datasets with many missing values (Fig 2b, Figure E in S1 File).

## Conclusion

CloudForest is a high-performance, extensible, and feature-rich implementation of the Random Forest algorithm. It supports classification and regression directly on common data types, such as binary, numerical and categorical features. Custom modification of the algorithm and data handling is provided through a set of general programmatic interfaces.

CloudForest is an open source project released under a three clause BSD-style license. The stable implementation released as part of this publication is found here: https://github.com/IlyaLab/CloudForest.

## Supporting Information

S1 File Supplementary text and figures. (PDF)
