# Supplementary material for: CloudForest: A Scalable and Efficient Random Forest Implementation for Biological Data
Source: PLoS One. 2015 Dec 17;10(12):e0144820. doi: 10.1371/journal.pone.0144820 (PMC4692062; doi:10.1371/journal.pone.0144820)
Supplement: S1 File — (PDF) [file pone.0144820.s001.pdf]

S1 File - CloudForest Supplementary Material

Contents

Documentation 1

Experimental details 2

For Figure 1 and Figure A 2

For Figure B 2

For Figure C 2

For Figure 2 and Figures D and E 3

Code snippets comparing CloudForest and SciKit-Learn 4

Citations 6

Supplementary Figure Legends 7

Documentation

Extensive documentation on CloudForest is found on the GitHub page: https://github.com/IlyaLab/CloudForest. The documentation includes installation instructions (https://github.com/IlyaLab/CloudForest#installation), guide lines for a quick start (https://github.com/IlyaLab/CloudForest#quick-start), and detailed explanations of all CloudForest functionalities.

The methods that apply to the features in the Random Forest, i.e. the features in general, but also methods specifically for numerical, categorical and target features are found here: https://github.com/ilyalab/CloudForest/blob/master/featureinterfaces.go. GoDoc documentation, which provides easy and structured access to all CloudForest functions, is found here: http://godoc.org/github.com/IlyaLab/CloudForest.

The original and continuously updated code repository is found here: https://github.com/ryanbressler/CloudForest.

Experimental details

For Figure 1 and Figure A

For all Random Forest (RF) implementations employed in Figure 1 and Figure A, we used the same standard settings. Specifically, the RFs consisted of 500 trees, the number features considered at each split was the square root of the total number of features, and the minimum number of samples per leaf was one. The experiments were run on a MacBookPro10,1, OS X 10.8.5, 2.8 GHz Intel Core i7 Ivy Bridge (3635QM), 8 GB 1600 MHz DDR3. The clinical feature matrix used for these experiments is found here https://github.com/IlyaLab/CloudForest/blob/master/data/clin.fm.

For Figure B

The CloudForest tests were run with the same settings as for Figure 1 and Figure A, except for the various extensions (roughly balanced bagging, etc.) described in the figure. This experiment was run on a compute server with eight cores (Intel Xeon CPU X5472 3.00 GHz). The number of cores (the number of jobs to run in parallel) was set to 8. An example of a CloudForest command for such an extension is given below:

```
growforest -train train_1.fm -target C:0 -nTrees 500 -rfpred rf_8_1.sf -ace 10 -cutoff .05 -
balance=true -nCores 8
applyforest -fm test_1.fm -rfpred rf_8_1.sf -preds rf_8_1.cl
```

Train_1.fm and Test_1.fm are training and test sets created with the nfold_utility (https://github.com/IlyaLab/CloudForest#nfold-utility) from the clinical feature matrix clin.fm.

For Figure C

CloudForest and SciKit-Learn's RandomForestClassifier were run on three datasets from the LIBSVM repository [1]:

Dataset	Citation	Number of classes	Number of training samples	Number of test samples	Number of features
Leukemia	[2]	2	38	34	7129
Gisette	[3]	2	6,000	1,000	5,000
Poker	[4]	10	25,010	1,000,000	10

Each dataset consists of a training set and a test set. RFs were trained on the training sets. For both implementations we used the same settings. Specifically, the RFs consisted of 5000 trees, the number features considered at each split was the square root of the total number of features, and the minimum number of samples per leaf was one. The number of cores (the number of jobs to run in parallel) was set to 8 for both implementations. The error was computed on the test set, and was calculated as the number of misclassified samples divided by the total number of samples. The experiment was run on a compute server with eight cores (Intel Xeon CPU X5472 3.00 GHz). An example for the CloudForest and SciKit-Learn commands for these different runs is given below. (The python wrapper sklrf.py is found here https://github.com/IlyaLab/CloudForest/blob/master/wrappers/python/sklrf.py).

```
growforest -train leu.libsvm -nTrees 5000 -rfpred cf_1_1.sf -target 0 -nCores 8 > cf1_1.time
applyforest -fm leu.t.libsvm -rfpred cf_1_1.sf -preds cf_1_1.cl
python sklrf.py leu.libsvm 5000 8 leu.t.libsvm skl1.cl > skl1.time
```

The .limsvm extensions were added to the original (extension-less) files, as CloudForest recognizes .libsvm files based on the extension (see https://github.com/IlyaLab/CloudForest#data-file-formats).

For Figure 2 and Figures D and E

CloudForest and SciKit-Learn's RandomForestClassifier were run on six datasets from the TCGA:

Dataset	Cit.	Number of samples	Number of P53 mutants (positive samples)	Number of features				Type of features		
				Total	Copy number	Mutation	Gene expression	Binary	Categorical	Numerical
BLCA	[5]	123	62	21948	3836	1627	16485	1627	70	20251
CRC	[6]	209	108	22607	106	1998	20503	1998	0	20609
GBM	[7]	144	48	28006	4350	8256	15400	8256	0	19750
HNSC	[8]	273	198	21280	3273	1800	16207	1800	66	19414
LUAD	[9]	466	251	36091	3621	17070	15400	17070	0	19021
STAD	[10]	255	116	34792	4296	8219	22277	8219	81	26492

Half of training samples were (randomly) selected for training and the other samples were used for testing. RFs were trained on the training sets. For both implementations we used the same settings. Specifically, the RFs consisted of 10,000 trees, the number features considered at each split was the square root of the total number of features, and the minimum number of samples per leaf was one. The number of cores (the number of jobs to run in parallel) was set to 12 for both implementations. The error was computed on the test set, and was calculated as the number of misclassified samples divided by the total number of samples. The experiment was run on a compute server with 16 cores (Intel Core Quad CPU Q8400 2.66GHz).

To impute missing values in the dataset (which originally contained no missing values) we employed the following strategy: (1) We computed the Pearson correlation coefficient and accompanying P-value for each feature with target, i.e. the binary mutation status of the tumor suppressor gene *TP53*. All features with a P-value<0.05 were identified as informative features. (2) The informative features were grouped into clusters using hierarchical clustering with correlation as a distance metric, complete linkage and a cutoff of 0.7. Thus, all pairs of features in a cluster had a Pearson correlation coefficient of 0.7 or higher. Each cluster was assigned an integer score, which as defined as the rounded -10log P-value of the feature with the smallest P-value in the cluster. (3) A cluster was randomly selected, where the probability of selecting a cluster was proportional to its integer score. Thus, clusters with highly informative features were more likely to be selected. After a cluster was selected, a sample in the training set was randomly selected, and the feature values of all features in that cluster for that sample were set to missing values ('NA'). This procedure was repeated until the proportion of missing values in the informative features across all training samples was 0%, 1%, 5%, 10%, 25% and 50%.

Code snippets comparing CloudForest and SciKit-Learn

For purposes of comparison, below we give the code for the CloudForest and SciKit-Learn implementation for impurity computation based on entropy.

https://github.com/ryanbressler/CloudForest/blob/master/entropytarget.go

```
package CloudForest
import (
	"math"
)
/*
EntropyTarget wraps a categorical feature for use in entropy driven classification
as in Ross Quinlan's ID3 (Iterative Dichotomizer 3).
*/
type EntropyTarget struct {
	CatFeature
}
//NewEntropyTarget creates a RefretTarget and initializes EntropyTarget.Costs to the proper
length.
func NewEntropyTarget(f CatFeature) *EntropyTarget {
	return &EntropyTarget{f}
}
/*
EntropyTarget.SplitImpurity is a version of Split Impurity that calls EntropyTarget.Impurity
*/
func (target *EntropyTarget) SplitImpurity(l *[]int, r *[]int, m *[]int, allocs
*BestSplitAllocs) (impurityDecrease float64) {
	nl := float64(len(*l))
	nr := float64(len(*r))
	nm := 0.0
	impurityDecrease = nl * target.Impurity(l, allocs.LCounter)
	impurityDecrease += nr * target.Impurity(r, allocs.RCounter)
	if m != nil && len(*m) > 0 {
		nm = float64(len(*m))
		impurityDecrease += nm * target.Impurity(m, allocs.Counter)
	}
	impurityDecrease /= nl + nr + nm
	Return
}
//UpdateSImpFromAllocs willl be called when splits are being built by moving cases from r to l
as in learning from numerical variables.
//Here it just wraps SplitImpurity but it can be implemented to provide further optimization.
func (target *EntropyTarget) UpdateSImpFromAllocs(l *[]int, r *[]int, m *[]int, allocs
*BestSplitAllocs, movedRtoL *[]int) (impurityDecrease float64) {
	target.MoveCountsRtoL(allocs, movedRtoL)
	nl := float64(len(*l))
	nr := float64(len(*r))
	nm := 0.0
	impurityDecrease = nl * target.ImpFromCounts(len(*l), allocs.LCounter)
	impurityDecrease += nr * target.ImpFromCounts(len(*r), allocs.RCounter)
	if m != nil && len(*m) > 0 {
		nm = float64(len(*m))
		impurityDecrease += nm * target.ImpFromCounts(len(*m), allocs.Counter)
	}
	impurityDecrease /= nl + nr + nm
	Return
}
func (target *EntropyTarget) ImpFromCounts(total int, counts *[]int) (e float64) {
	p := 0.0
```

```
        for _, i := range *counts {
                if i > 0 {
                        p = float64(i) / float64(total)
                        e -= p * math.Log(p)
                }
        }
        return
}
//EntropyTarget.Impurity implements categorical entropy as sum(pj*log2(pj)) where pj
//is the number of cases with the j'th category over the total number of cases.
func (target *EntropyTarget) Impurity(cases *[]int, counts *[]int) (e float64) {
        total := len(*cases)
        target.CountPerCat(cases, counts)
        p := 0.0
        for _, i := range *counts {
                if i > 0 {
                        p = float64(i) / float64(total)
                        e -= p * math.Log(p)
                }
        }
        return
}
```

https://github.com/scikit-learn/scikit-learn/blob/master/sklearn/tree/_tree.pyx#L368

```
cdef class Entropy(ClassificationCriterion):
    """Cross Entropy impurity criteria.
    Let the target be a classification outcome taking values in 0, 1, ..., K-1.
    If node m represents a region Rm with Nm observations, then let
        pmk = 1/ Nm \sum_{x_i in Rm} I(yi = k)
    be the proportion of class k observations in node m.
    The cross-entropy is then defined as
        cross-entropy = - \sum_{k=0}^{K-1} pmk log(pmk)
    """
    cdef double node_impurity(self) nogil:
        """Evaluate the impurity of the current node, i.e. the impurity of
           samples[start:end]."""
        cdef double weighted_n_node_samples = self.weighted_n_node_samples
        cdef SIZE_t n_outputs = self.n_outputs
        cdef SIZE_t* n_classes = self.n_classes
        cdef SIZE_t label_count_stride = self.label_count_stride
        cdef double* label_count_total = self.label_count_total
        cdef double entropy = 0.0
        cdef double total = 0.0
        cdef double tmp
        cdef SIZE_t k
        cdef SIZE_t c
        for k in range(n_outputs):
            entropy = 0.0
            for c in range(n_classes[k]):
                tmp = label_count_total[c]
                if tmp > 0.0:
                    tmp /= weighted_n_node_samples
                    entropy -= tmp * log(tmp)
            total += entropy
            label_count_total += label_count_stride
        return total / n_outputs
    cdef void children_impurity(self, double* impurity_left,
                                double* impurity_right) nogil:
```

```
        """Evaluate the impurity in children nodes, i.e. the impurity of the
           left child (samples[start:pos]) and the impurity the right child
           (samples[pos:end])."""
        cdef double weighted_n_node_samples = self.weighted_n_node_samples
        cdef double weighted_n_left = self.weighted_n_left
        cdef double weighted_n_right = self.weighted_n_right
        cdef SIZE_t n_outputs = self.n_outputs
        cdef SIZE_t* n_classes = self.n_classes
        cdef SIZE_t label_count_stride = self.label_count_stride
        cdef double* label_count_left = self.label_count_left
        cdef double* label_count_right = self.label_count_right
        cdef double entropy_left = 0.0
        cdef double entropy_right = 0.0
        cdef double total_left = 0.0
        cdef double total_right = 0.0
        cdef double tmp
        cdef SIZE_t k
        cdef SIZE_t c
        for k in range(n_outputs):
            entropy_left = 0.0
            entropy_right = 0.0
            for c in range(n_classes[k]):
                tmp = label_count_left[c]
                if tmp > 0.0:
                    tmp /= weighted_n_left
                    entropy_left -= tmp * log(tmp)
                tmp = label_count_right[c]
                if tmp > 0.0:
                    tmp /= weighted_n_right
                    entropy_right -= tmp * log(tmp)
            total_left += entropy_left
            total_right += entropy_right
            label_count_left += label_count_stride
            label_count_right += label_count_stride
        impurity_left[0] = total_left / n_outputs
        impurity_right[0] = total_right / n_outputs
```

Citations

1. Chang, C.-C. and C.-J. Lin, *LIBSVM: a library for support vector machines.* ACM Transactions on Intelligent Systems and Technology (TIST), 2011. 2(3): p. 27.

2. Golub, T.R., et al., *Molecular classification of cancer: class discovery and class prediction by gene expression monitoring.* science, 1999. 286(5439): p. 531-537.

3. Guyon, I., et al. *Result analysis of the nips 2003 feature selection challenge*. in *Advances in Neural Information Processing Systems*. 2004.

4. Bache, K. and M. Lichman, *UCI machine learning repository.* URL http://archive.ics.uci.edu/ml, 2013. 19.

5. Network, C.G.A.R., *Comprehensive molecular characterization of urothelial bladder carcinoma.* Nature, 2014. 507(7492): p. 315-322.

6. Network, C.G.A., *Comprehensive molecular characterization of human colon and rectal cancer.* Nature, 2012. 487(7407): p. 330-337.

7. Brennan, C.W., et al., *The somatic genomic landscape of glioblastoma.* Cell, 2013. 155(2): p. 462-477.

8. Network, C.G.A., *Comprehensive genomic characterization of head and neck squamous cell carcinomas.* Nature, 2015. 517(7536): p. 576-582.

9. Network, C.G.A.R., *Comprehensive molecular profiling of lung adenocarcinoma.* Nature, 2014. 511(7511): p. 543-550.

10. Network, C.G.A.R., *Comprehensive molecular characterization of gastric adenocarcinoma.* Nature, 2014.

Supplementary Figure Legends

Figure A | Expanded version of Figure 1, which also includes results for RF-ace, and for CloudForest applied to the datasets after categorical features have been transformed into binary features using one-hot encoding (CF on bin. cat. data).

Figure B | Classification performance of CloudForest on the clinical dataset with various extensions.

Figure C | Classification performance (top) and training time (bottom) for SciKit-Learn's RandomForestClassifier (SKL) and CloudForest with and without various extensions on three LIBSVM datasets; leukemia (left), gisette (middle) and poker (right).

Figure D | Comparison between CloudForest and SkiKit-Learn in terms of prediction performance for six TCGA datasets with varying numbers of missing values (x-axis).

Figure E | Comparison between CloudForest and SkiKit-Learn in terms of computation time for six TCGA datasets with varying numbers of missing values (x-axis).

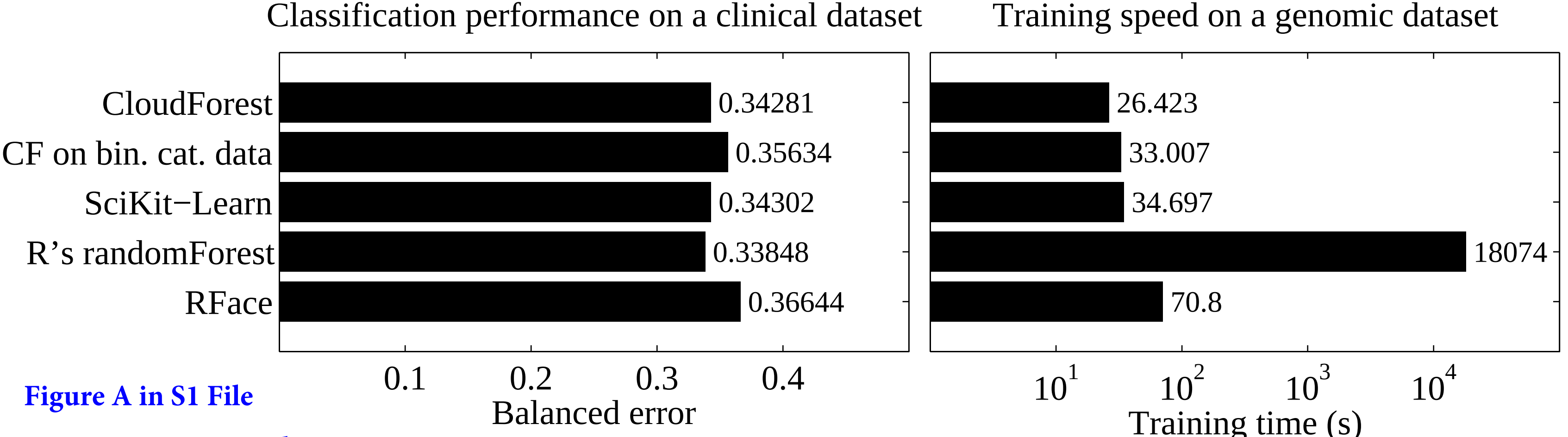

Figure A in S1 File

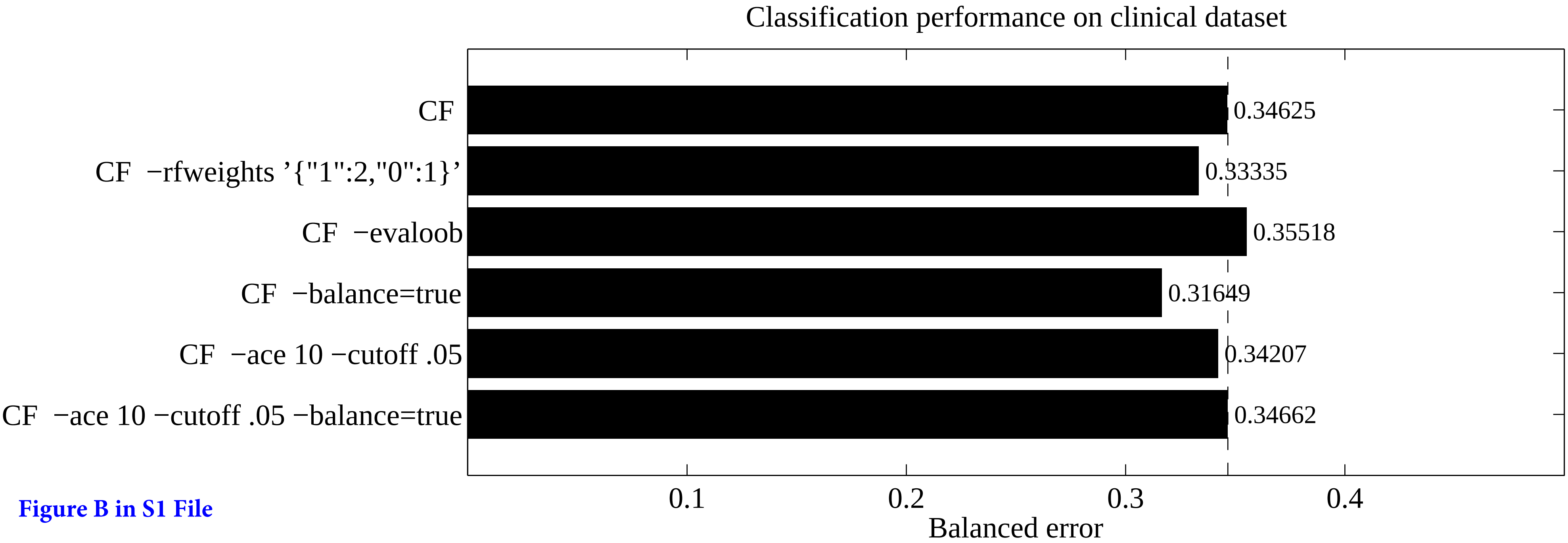

Figure B in S1 File

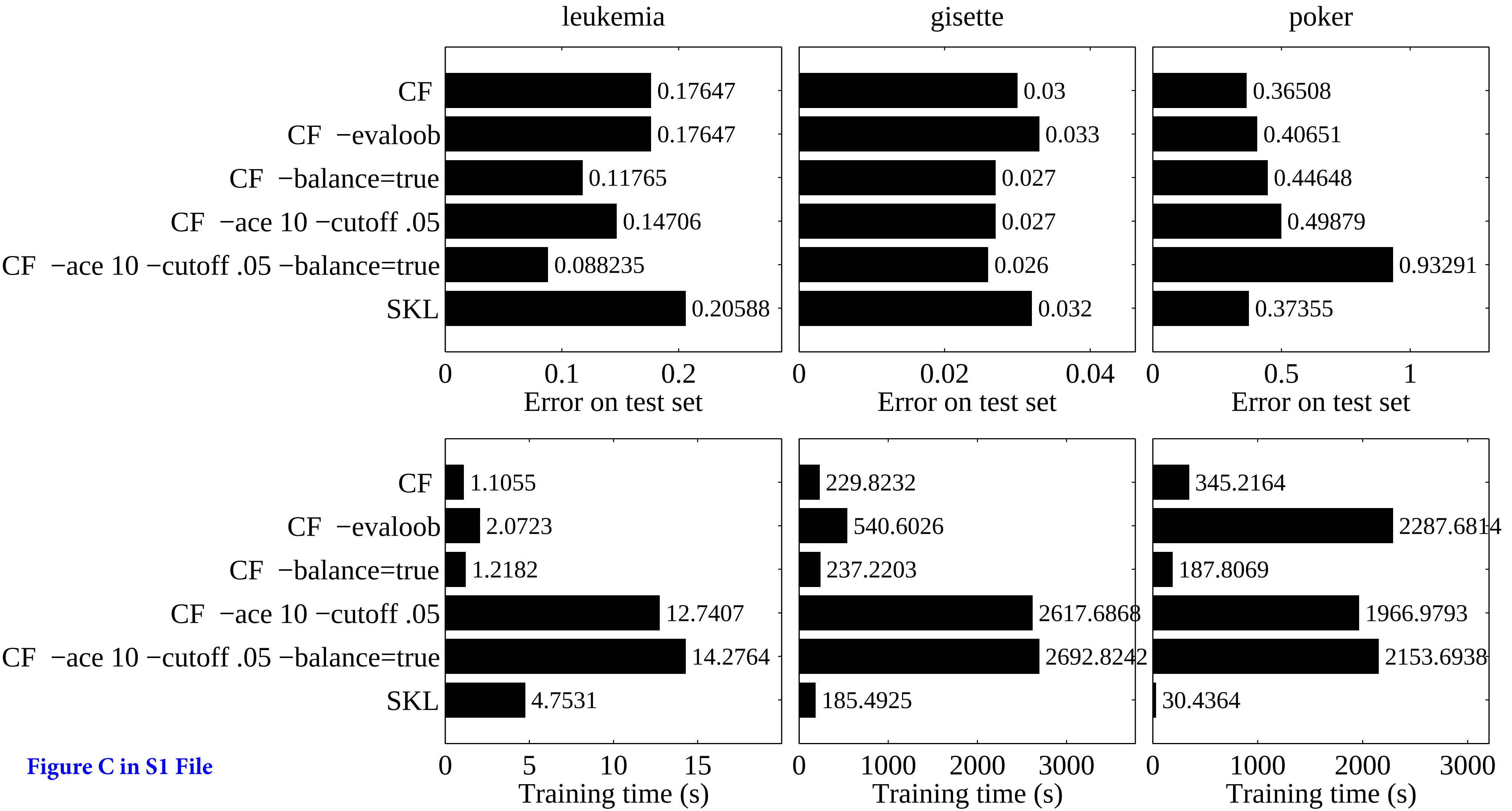

Figure C in S1 File

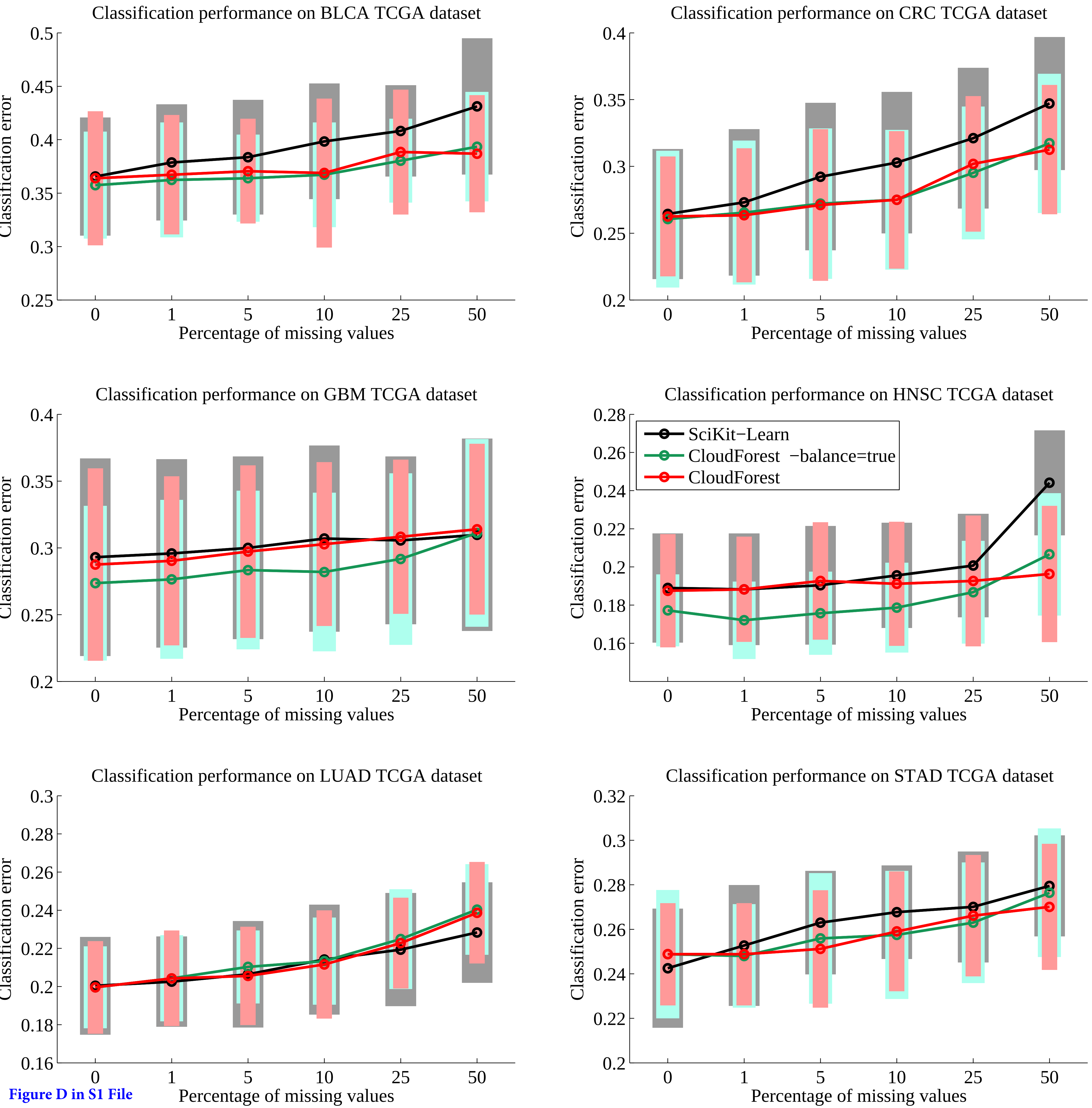

Figure D in S1 File

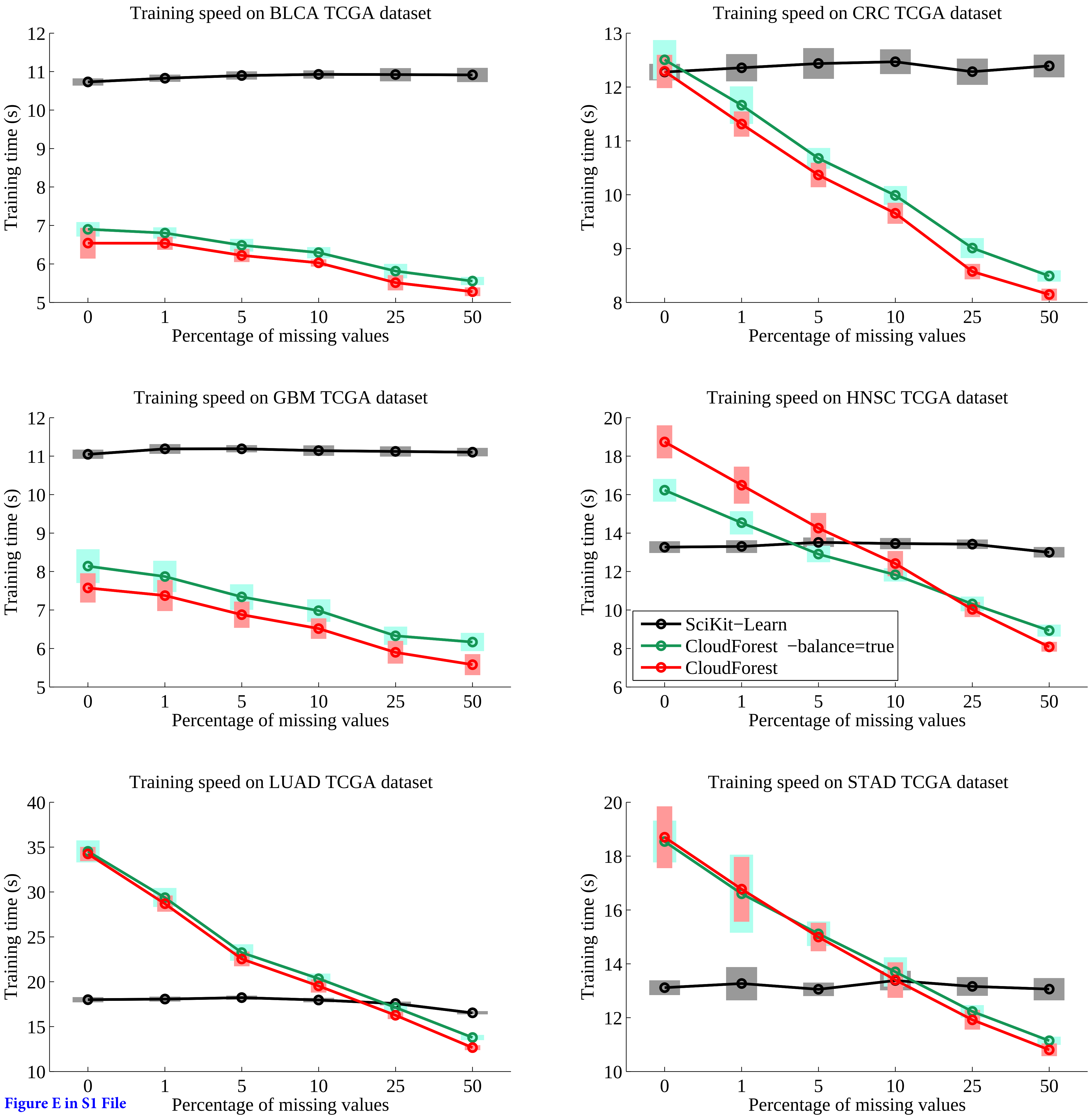

Figure E in S1 File
